# Deforestation reduces rainfall and agricultural revenues in the Brazilian Amazon

**DOI:** 10.1038/s41467-021-22840-7

**Published:** 2021-05-10

**Authors:** Argemiro Teixeira Leite-Filho, Britaldo Silveira Soares-Filho, Juliana Leroy Davis, Gabriel Medeiros Abrahão, Jan Börner

**Affiliations:** 1 Centre for Remote Sensing, Federal University of Minas Gerais, Belo Horizonte, Brazil; 2 Department of Agricultural Engineering, Federal University of Viçosa, Viçosa, Brazil; 3 Centre for Development Research, University of Bonn, Bonn, Germany

**Keywords:** Attribution, Governance, Environmental impact

## Abstract

It has been suggested that rainfall in the Amazon decreases if forest loss exceeds some threshold, but the specific value of this threshold remains uncertain. Here, we investigate the relationship between historical deforestation and rainfall at different geographical scales across the Southern Brazilian Amazon (SBA). We also assess impacts of deforestation policy scenarios on the region’s agriculture. Forest loss of up to 55–60% within 28 km grid cells enhances rainfall, but further deforestation reduces rainfall precipitously. This threshold is lower at larger scales (45–50% at 56 km and 25–30% at 112 km grid cells), while rainfall decreases linearly within 224 km grid cells. Widespread deforestation results in a hydrological and economic negative-sum game, because lower rainfall and agricultural productivity at larger scales outdo local gains. Under a weak governance scenario, SBA may lose 56% of its forests by 2050. Reducing deforestation prevents agricultural losses in SBA up to US$ 1 billion annually.

## Abstract (web summary)

Deforestation in the Amazon region has suggested to influence precipitation in a non-linear way. Here, the authors show that forest loss is associated with decreasing precipitation after a scale-dependent threshold is crossed, which can cause stress on agriculture if deforestation is expanded.

## Introduction

**Fig. 1:**
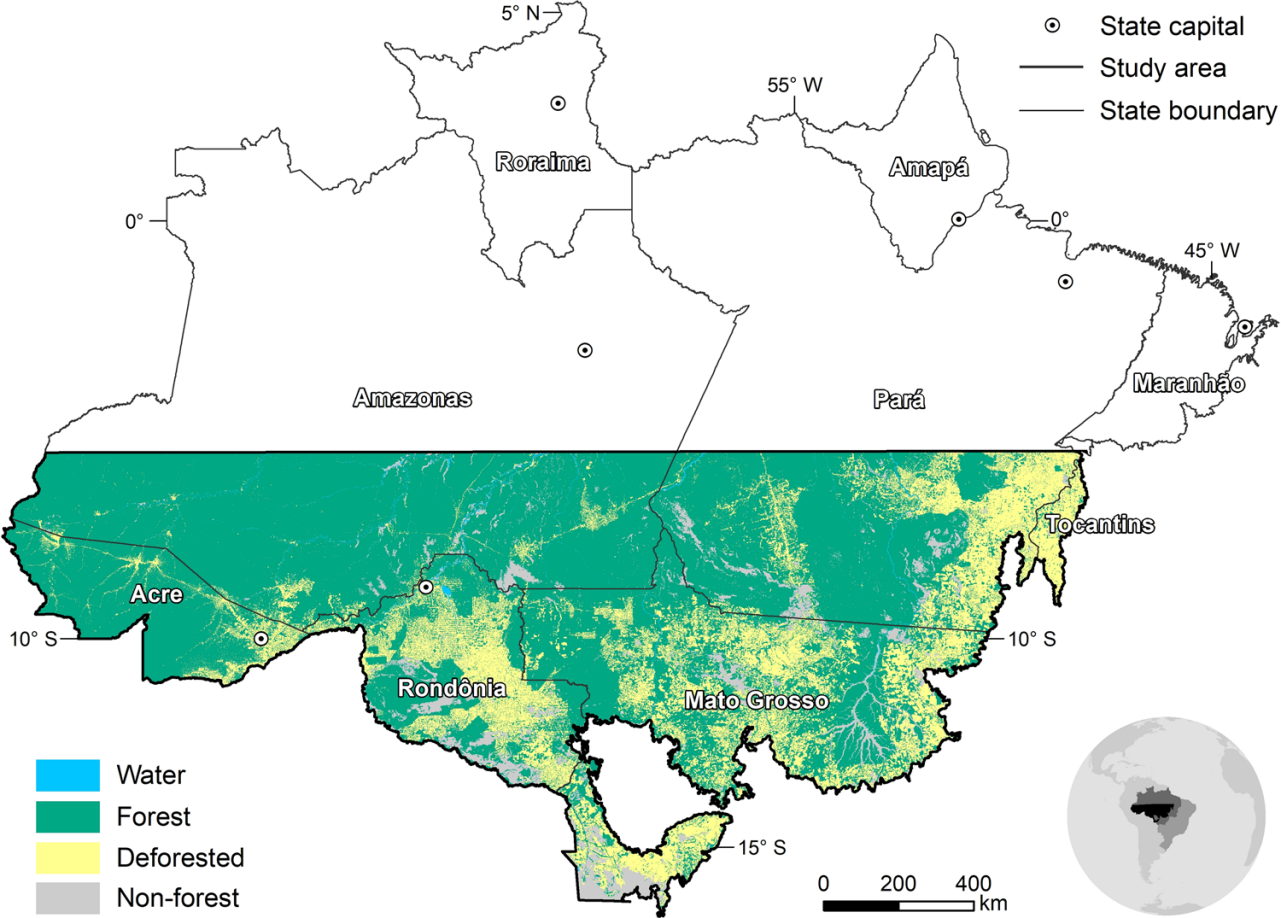
Land cover in the Southern Brazilian Amazon as of 2019. Southern Brazilian Amazon encompasses 1.9 million km² of the Amazon biome. Deforested area by 2019 according to data from the Program to Calculate Deforestation in the Amazon (PRODES).

Mounting evidence from model-based and empirical research indicates that the Amazon forest influences the spatiotemporal patterns and amount of rainfall^1–4^. Among the multiple forest ecosystem services, rainfall regulation is key to sustain agriculture in the region and beyond^5^, where an important share of Brazil’s soy and beef is produced. Despite this well-documented function, deforestation in the Brazilian Amazon is on the rise again. As of 2020, PRODES^6^ reported 11,000 km^2^ of forest loss, a 143% increase from 2012, the lowest deforestation rate on record. As forest loss accumulates, impacts on rainfall patterns may critically affect agriculture, especially in the Southern Brazilian Amazon (SBA), where forest losses already amount to 30%^6^. SBA is one of the most dynamic land-use frontiers in the world^7^ and accounts for the lion’s share of croplands and pastures in the Brazilian Amazon (Fig. 1).

Despite extensive research on greenhouse gas (GHG)-induced climate change^8^, few studies have addressed regional climate change in response to extensive land use and cover change (LUCC), such as deforestation. Forest conversion to pasture and croplands (forest loss) affects moisture cycling and energy balance^9,10^ and may change rainfall patterns^3,4,11,12^. Hence, the regional climate could respond as much as or even stronger to LUCC than to global warming^13,14^. In the Amazon, the estimated impact on the annual radiative budget due to surface albedo-change is approximately six times higher than that of aerosol emissions, and projected impacts on agriculture from future deforestation-induced changes in climate are of the same magnitude as those from global climate change under the RCP 8.5 scenario^15^.

Studies point to a reliance of the Amazon regional rainfall regimes on the forest^16–18^. Although the effects of biome-wide deforestation affecting forest moisture recycling and irreversible biome transition are still relatively uncertain^19–21^, at smaller geographical scales, some studies suggest a negative linear response of rainfall to forest loss^22–24^, while others indicate a nonlinear response^2,25–27^. According to the latter, small-scale, patchy, heterogeneous deforestation patterns drive changes in mesoscale circulation of moisture flow from forests into the atmosphere that can enhance precipitation over nearby deforested areas^20,27–29^. However, as forest loss progresses, the region eventually reaches a critical threshold beyond which this relationship reverses with additional forest loss rapidly reducing rainfall^9,25,30,31^.

At regional scales (≈200–600-km grid-cell size), modelling experiments indicate that the critical threshold of forest loss beyond which rainfall progressively decreases lies between 30 and 50% of forest loss, depending on the spatial patterns of deforestation^25,30^. But to date, no study has empirically identified this critical threshold and the consequences of crossing it by using observed rainfall data.

In this work, we fill this gap by detrending the effects of geographic location, elevation and interannual variability on a satellite-derived rainfall time series to correlate the residual anomalies with forest loss at different geographical scales (28–224-km grid-cell sizes). To gauge future critical thresholds of forest loss and associated agricultural economic losses, we use projections of agricultural expansion in the region under weak and strong environmental governance scenarios and estimates of soybean and pasture yield losses due to rainfall reduction caused by deforestation across the Brazilian Amazon.

## Results and discussion

### Relationship between deforestation and rainfall at different geographical scales

**Fig. 2:**
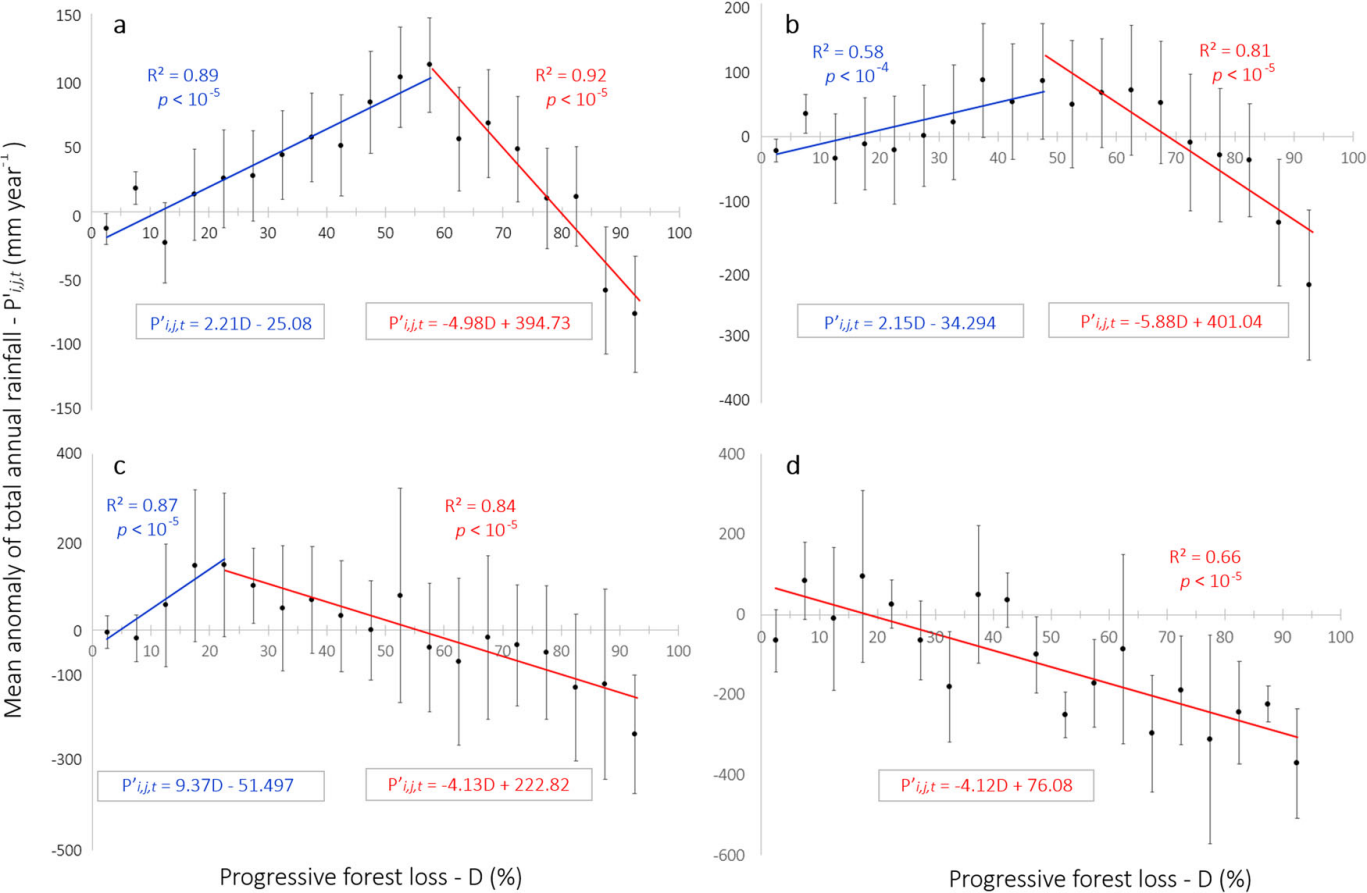
Mean annual rainfall anomalies per forest loss percent within 28-, 56-, 112- and 224-km grid cells. Two piecewise linear segments from MARS algorithm: **a** *D* < 57.5% (blue line) and *D* > = 57.5% (red line) for 28-km grid cells. **b** *D* < 47.5% (blue line) and *D* > = 47.5% (red line) for 56-km grid cells. **c** *D* < 27.5% (blue line) and *D* > = 27.5% (red line) for 112-km grid cells. **d** Best-fit linear model (dashed red line) for 224-km grid cells. Error bar represents the standard error of the mean rainfall anomaly for each forest loss interval. P*’*_*i,j,t*_ are the residual annual rainfall anomalies (in mm/year), where the subscripts *i* and *j* represent space dimensions and the subscript *t* represents time dimension. *D* represents the progressive forest loss fraction (in percentage).

The relationship between forest loss fractions and annual mean rainfall anomalies at 28-, 56- and 112-km grid cells cannot be explained by using a single linear regression; the best fit was obtained by adjusting a multivariate adaptive regression spline (MARS) composed of two piecewise linear segments (Fig. 2a–f). In turn, the best fit for the 224 × 224-km grid cells was attained by a single linear regression.

Forest loss affects rainfall differently, depending upon the geographical scale (Fig. 2a–d). At the original spatial resolution of the TRMM data (28 × 28-km grid cell), there is a dual effect of forest loss, with rainfall increasing with forest loss up to ≈58% and then decreasing afterwards (Fig. 2a). The gain before the critical threshold is of +22.1 ± 9.3 mm of precipitation per 10% of additional forest loss. However, beyond 57.5% of forest loss, precipitation reduces precipitously, with each 10% of additional forest loss inducing a net reduction in annual rainfall of −49.2 ± 11.3 mm.

We tested for correlation between forest loss and the mean long‐term trend in rainfall using *t* tests (Supplementary Section 1.4). The test comparing the annual rainfall between cells with smaller forest loss and cells that experienced larger forest loss within two separated periods: 1999–2009 and 2010–2019 (Supplementary Table 6) demonstrated that the cells with smaller historical forest loss (below 55–60%) experienced an increase in annual rainfall by +96.9 ± 12.65 mm. Conversely, cells with larger forest losses (exceeding 55–60%) exhibited an annual decrease of −306.4 ± 42.77 mm (Supplementary Table 7). Both statistical inferences were statistically significant with *P* < 0.05.

Aggregating both forest loss and rainfall data to a coarser scale (56 × 56-km grid cell), the critical threshold dropped to ≈48% (Fig. 2b). For 112 × 112-km grid cells, both the positive and negative effects were attenuated and the threshold reduced even more to ≈23% of forest loss (Fig. 2c). This dual response of rainfall to forest loss is consistent with the theory and climate modelling experiments^26,30^. At smaller geographical scales (28 and 56 km), the reduction in rainfall after the critical threshold was more than twice as fast as the increase before the threshold. However, at larger geographical scales, this effect attenuated until we found a constant linear reduction at 224 × 224-km grid cells (Fig. 2d).

Our multiscale analysis supports the argument that, if neighbouring grid cells also have high levels of forest loss, the critical threshold of forest loss kicks in at a lower level of forest loss. Therefore, widespread deforestation results in a negative-sum game where total reduction in rainfall outdoes local gains. In this sense, the critical thresholds we found within smaller grid cells are conservative because as regional deforestation progresses, local gains diminish and eventually cancel out.

At the scale from 28 to 56 km, the land-cover heterogeneity forms anomalous meteorological vertical cells, with enhanced convection and hence increased rainfall over part of the deforested patch and rainfall suppression over the surrounding forested regions, what is known as rainfall dipoles^20^. The location, size, strength and net effect of the rainfall dipoles are heavily influenced by the size of the deforested patch, large-scale winds and surrounding forested area^20,31^.

On the other hand, the reduction in rainfall at a larger geographical scale (224 × 224 km) is caused by a combination of the higher albedo of deforested surfaces and the smaller year-round evapotranspiration of crops and pastures relative to natural vegetation^27^. The former effects in addition to reduced surface roughness, which in turn diminishes the vertical transport of turbulence by horizontal winds^27,32,33^, decrease atmospheric moisture available for precipitation. In this respect, Sena et al.^15^ indicate that precipitable water above deforested areas is ≈7% lower than that above the Amazon forest. Thus, the critical threshold is the point after which the combined effects of the higher albedo, smaller evapotranspiration and reduced surface roughness due to forest loss become stronger than the deforestation breeze effect, which weakens with forest loss. The net reduction we find after the critical threshold is much steeper than the prior net gains, which is consistent with climate modelling results^27,31^.

The quantitative effects of forest loss on rainfall are for the study region as a whole. Particular grid cells may be more or less affected according to their locations. The smallest interannual variability is verified in grid cells located in the south and southwest of the Amazon (Supplementary Fig. 7), where the annual rainfall is lower (Supplementary Fig. 1). Consequently, rainfall reduction due to forest loss is proportionally larger in those regions, currently reaching up 48% (Supplementary Fig. 8). This has economic implications for agriculture since the areas most affected by rainfall reduction are major soy production zones or regions where agriculture is likely to expand in the future (Supplementary Fig. 9). Crop yields and the success of double-cropping systems already vary significantly from year to year in SBA because of the interannual climate variability^34,35^. The effect of forest loss on rainfall superimposed to the interannual variability thus poses an additional risk of crop failure in dry or drought years, which tend to become more frequent and intense as deforestation progresses alongside climate change^36^ (Supplementary Section 2.1).

### Additional evidence for forest loss causation of rainfall reduction

The relationship between forest loss and rainfall presented here is correlational. To demonstrate that this relationship is significantly greater than a potentially reverse causal effect, i.e., more severe deforestation systematically taking place in drier (or drying) parts of the forest, we superimposed a map of the effects of Maximum Climatological Water Deficit on deforestation^36,37^ over our study region. We found that only 12 cells of a total 86 non-null cells (14% of our study region) showed a statistically significant effect that more severe forest loss tends to take place in drier (or drying) parts (Supplementary Fig. 11). However, the signal in these few grid cells was near zero. Although the feedback between drought and forest loss is notable in more humid regions of the Amazon that lack a marked dry season^37^, this is not the case for SBA, where the dry season is longer than 150 days^38^, hence sufficiently long to dry out the chopped-down understory vegetation to completely clear-cut the forest. In addition, empirical evidence suggests that the length of the rainy season decreases ≈0.9 ± 0.34 days for each additional 10% of forest loss^4^ (Supplementary Section 2.2).

Finally, we measured the spatial association between rainfall anomalies and forest loss by calculating the Cramer’s V^39^ and the Spearman Rank Order Correlation coefficient^40^. After detrending the other signals, the resulting anomalies exhibited a spatial variability that correlated only with forest loss (Supplementary Fig. 10), which corroborates the hypothesized causal direction from forest loss to rainfall reduction in SBA (Supplementary Section 2.2).

### Future critical forest loss thresholds and associated agricultural economic losses

To assess the impacts of future regional rainfall reduction on agriculture, we simulated deforestation across SBA. To do so, we ran Otimizagro^41,42^, a countrywide LUCC model for Brazil, from 2015 to 2050 under two alternative policy scenarios, i.e., weak and strong environmental governance^42^ (Supplementary Section 1.5). The weak governance scenario (WEG) assumed the continued dismantling of Brazil’s conservation policies along with strong political support for environmentally damaging agricultural practices and implicit economic incentives for illegal deforestation (Supplementary Section 1.5.1). The strong governance (SEG) scenario incorporated effective enforcement of conservation policies based on sustained political support for the environmental agenda in Brazil, including the full implementation of the Forest Code and additional conservation incentives^42,43^ (Supplementary Section 1.5.2).

**Fig. 3:**
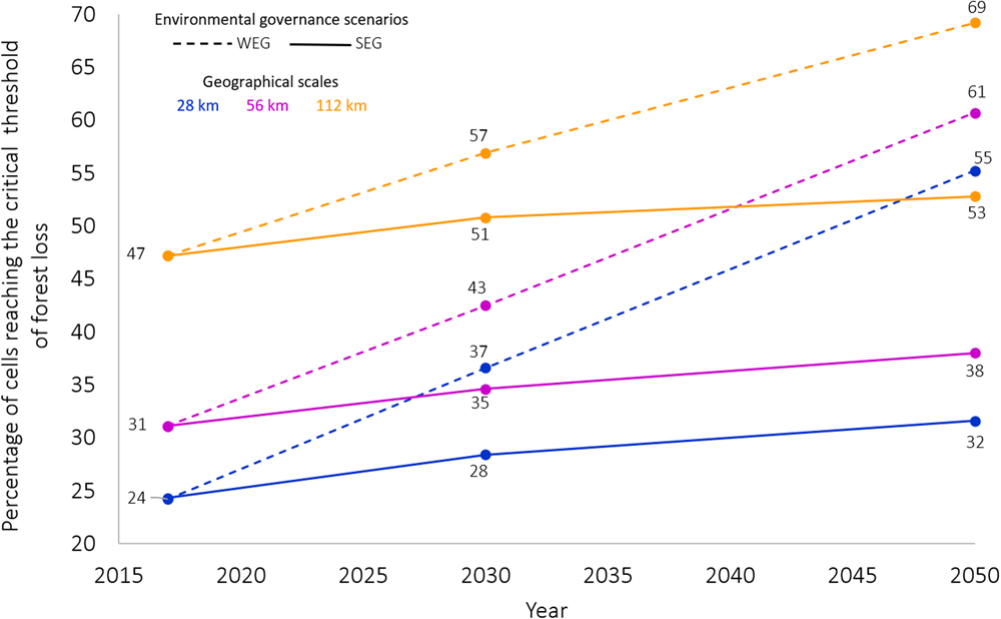
Percentage of different grid-cell sizes crossing the critical threshold. Percentage of different grid-cell sizes crossing the critical threshold of forest loss in southern Brazilian Amazon over time under the two environmental governance scenarios: weak scenario (WEG) and strong scenario (SEG) within 28-km, 56-km and 112-km grid cells.

As of 2019, 25% of SBA had already reached the 55–60% threshold of forest loss within 28 × 28-km grid cells. This critical threshold under the WEG would occur in 36% and 55% of the region’s grid cells by 2030 and 2050, respectively (Fig. 3). The realization of the SEG scenario could abate by 24% the number of grid cells that would reach the critical threshold by 2050 under the WEG. At coarser scales (56 km and 112 km), the critical threshold of forest loss (*D* ≥ 47.5% and *D* ≥ 27.5%) under the WEG would occur in 61% and 69% of the region’s grid cells by 2050, respectively. Again, the full implementation of the SEG scenario could abate by 28% and 16% the number of 56- and 112-km grid cells, respectively, that would reach the critical threshold of forest loss by 2050 under the WEG.

**Fig. 4:**
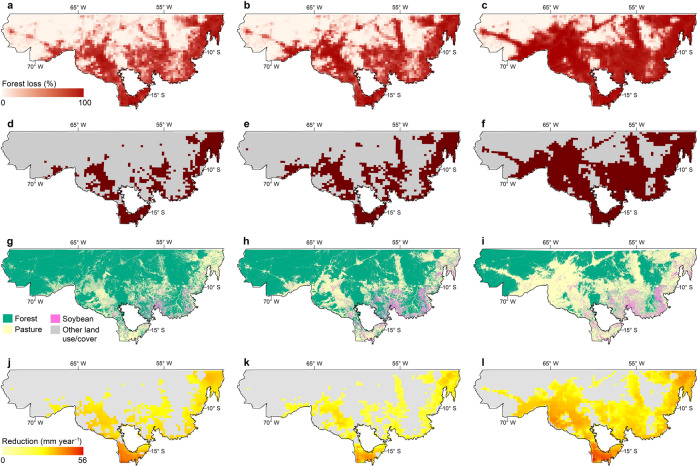
Percentage of forest loss, 28 × 28-km grid cells reaching the critical threshold, land use/cover and rainfall reduction. Percentage of forest loss: **a** by 2019. **b** Simulated for 2050 for SEG and **c** WEG. In all, 28 × 28-km grid cells reaching the critical forest loss threshold: **d** by 2019. **e** Simulated by 2050 for SEG. **f** WEG scenario. Land use/cover: **g** by 2019, **h** simulated by 2050 for SEG **i** and WEG. Rainfall reduction: **j** by 2019. Simulated by 2050 for **k** SEG and **l** WEG.

Similar to other tropical regions, agriculture in the Amazon is mostly rainfed^44^, and as such, decreases in annual rainfall in response to forest loss will reduce yields or shift agriculture either away from the region or towards more drought-resistant crops. Forest suppression also delays the onset of the rainy season^3,4,45^, prepones its end^4,45^ and shortens its length^4,46^. All of these rainy season characteristics are essential for the highly productive double-cropping systems in the region^34,35^. Hence, continued deforestation imposes major challenges for agricultural production in SBA, especially in regions that have already reached the critical threshold of forest loss within the 28 × 28-km grid cells, such as the north-eastern and south-eastern regions of Pará State, West of Maranhão State, the central part of Rondônia and, most notably, the northern soybean belt in Mato Grosso State (Fig. 4d–f).

To gauge the potential agricultural economic losses implied by our two scenarios, we used the projections of agricultural expansion in the region for the SEG and WEG scenarios from the Otimizagro model^42^ and estimates of soybean and pasture yield losses due to rainfall reduction caused by deforestation across the Brazilian Amazon^47^. Our goal was to explore whether the hydrological zero-sum game outlined above equivalently applies to the economics of agricultural expansion in the region. If so, the opportunity costs, in terms of foregone revenues from converting less forest to crop and pasturelands in the SEG scenario, would have to be outperformed by losses in crop and pasture productivity across SBA due to changes in rainfall patterns under the WEG (Supplementary Section 1.6).

Considering only revenues from soy cultivation and beef production in SBA, we find that productivity losses and associated revenues under WEG (US$ 5.6 billion for soy and US$ 180.8 billion for beef production by 2050 in net present values—NPV) dwarf the conservation opportunity costs of US$ 19.5 billion in NPV under SEG. In other words, Brazil may have passed a threshold at which further Amazon deforestation translates into direct economic damage. Deforestation does not only result in CO_2_ emissions and irreversible loss of globally valued biodiversity, it also imposes massive productivity losses worth up U$ 1 billion annually (Equivalent Annual Annuity; Supplementary Section 1.6) on the region’s agribusiness. It is unlikely, at least in the short or medium term, that accounting for adaptation costs and potential benefits would tip the balance in favour of agricultural expansion.

Anticipating the impacts of deforestation on the region’s ecosystem services, especially rainfall regulation for agriculture, is paramount to convince policymakers and other stakeholders to act before it is too late. Indeed, acknowledging those risks could help steer Brazil back to a course that sustainably integrates agricultural production and environmental conservation. Brazil’s agribusiness and their global partners are testing the limits of nature by expanding into natural forests at the risk of reducing the rainfall that sustains its productivity. The current land-use trajectory in the Brazilian Amazon, therefore, puts the largely rainfed agricultural systems of the country on an unsustainable pathway.

## Methods

We analyzed the quantitative linkage between annual rainfall and Amazon forest loss from 1999 to 2019. Using a multiscale approach, we empirically determined the level of forest loss (the critical threshold) beyond which the effect of forest loss on rainfall reverses. Our approach sheds light on the unresolved question as to how continued forest loss affects annual rainfall at different geographical scales. We also explored when and which regions in SBA may reach the critical threshold of forest loss under two environmental governance scenarios (SEG and WEG)^42^.

### Region

SBA covers 1.9 million km². This region historically underwent agricultural and logging expansion from the south and east of Brazil.

### Rainfall data

We used the rainfall data between 1999 and 2019 from the Tropical Rainfall Measuring Mission satellite (TRMM) 3B43 product (version 7). Monthly rainfall was aggregated to obtain yearly rainfall. TRMM data are originally at ≈28 × 28-km spatial resolution. This rainfall dataset has been extensively verified and validated for the Amazon biome^48,49^.

### Deforestation data

We used data from PRODES (Program to Calculate Deforestation in the Amazon)^6^, originally released at ≈30-m spatial resolution. We aggregated PRODES data into time-series maps of percentages of forest loss per grid-cell sizes from 28 × 28 km, 56 × 56, 112 × 112 to 224 × 224 km.

### Anomalies of annual rainfall

Rainfall in the region has a marked spatial gradient (Supplementary Fig. 1) and interannual variability. Thus, to minimize omitted variable bias in our analysis, we first removed the effects of factors other than deforestation that may affect rainfall across both time and space. To do so, we calculated anomalies of annual rainfall using a three-step procedure (Supplementary Fig. 4), based on a conceptual methodology developed for analyzing the onset, end and length of the rainy season in Southern Amazon^34^. We calculated anomalies of annual rainfall (P′_*i,j,t*_) using the three-step procedure summarized in Eqs. (1), (2) and (3) and Supplementary Fig. 4 (Supplementary Sections 1.1 and 1.2). Notations are as follows: *i, j, t* are the subscripts representing space (*i, j*) and time (*t*), ${{\bf{P}}}_{{\boldsymbol{i}},{\boldsymbol{j}},{\boldsymbol{t}}}$ are the annual rainfall values in mm, ${\hat{\bf{P}}}_{{\boldsymbol{i}},{\boldsymbol{j}}}$ are the estimated values of rainfall due to geographical location and elevation, ${\bf{P}}_{{\boldsymbol{i}},{\boldsymbol{j}},{\boldsymbol{t}}}$ is the difference between observed annual rainfall values and the estimated ones due to geographical location and elevation, ${\bar{{\bf{P}}}}_{{\boldsymbol{t}}}$ are the annual averages of rainfall calculated throughout the study region calculated from 1999 to 2019, ${{\bf{P}}}_{{\boldsymbol{i}},{\boldsymbol{j}},{\boldsymbol{t}}}^{\prime}$ are the residual annual rainfall anomalies and ${\boldsymbol{\varphi }},{\boldsymbol{\lambda }},{\boldsymbol{\zeta }}$ are latitude, longitude (in degrees) and elevation (in metres).

#### Step 1

To estimate the spatial pattern of annual rainfall, we used a second-degree model (*r*² = 0.72, *P* > 10^−5^) to compute estimated values of rainfall due to this geographical pattern so that

1 $${\hat{{\rm{P}}}}_{i.j}=	 -8340+\left(27.55\varphi \right)+\left(-1.359{\varphi }^{2}\right)+\left(-366.2\lambda \right)+\left(-3.005{\lambda }^{2}\right)\\ 	 +\left(-2.118\zeta \right)+(0.003{\zeta }^{2})$$

#### Step 2

To remove the climatological trend related to geographic location and elevation, we calculated the difference between raw values of observed rainfall in each year and the estimated values due to geographical position obtained from Eq. (1), so that

2 $${{\rm{P}}\ast }_{i.j.t}=\left({{\rm{P}}}_{i.j.t}-{\hat{{\rm{P}}}}_{i.j}\right)$$

#### Step 3

To remove the signal of large-scale factors, such as that of the ENSO, we subtracted the mean annual rainfall for the whole study region from the outputs of Eq. (2)

3 $${{\rm{P}}}_{I,j,t}^{{\prime} }={{\rm{P}}\ast }_{i.j.t}-{\bar{{\rm{P}}}}_{t}$$

The residual is assumed to be an “anomaly” that is not explained by the geographic location, elevation or large-scale time-varying factors.

### LUCC modelling and associated agricultural economic impact

Otimizagro is a spatially explicit model that simulates land use, land-use change, forestry, deforestation and regrowth under various scenarios of agricultural land demand and deforestation policies for Brazil^41^. The model simulates nine annual crops (including single and double cropping), five perennial crops, and plantation forests. The model framework, developed using the Dinamica EGO platform (dinamicaego.com), is structured in four spatial levels: (i) Brazil’s biomes, (ii) IBGE micro-regions, (iii) Brazilian municipalities and (iv) a raster grid of 25 ha spatial resolution. Future demand for crops and deforestation, and regrowth rates are exogenous to the model. We assumed constant agricultural practices and prices over time under both governance scenarios. Opportunity costs are calculated as follows:

(I) We performed a simulation of deforestation in the Brazilian Amazon, as well as soybean and pasture expansion under the SEG and WEG scenarios^42^. (II) We calculated the average productivity using regional estimates of soybean and pasture yield losses due to rainfall reduction caused by Amazon biome-wide deforestation^47^ (Supplementary Section 1.6). (III) We projected productivity change until 2050 adjusting future soybean yield projections (3.7 ton/hectare)^50^ and pasture productivity projections^51^ (2.9 arroba/hectare)^51^ by the average productivity losses calculated in step II. (IV) We computed annual revenues in US$ per hectare using current soybean and cattle arroba prices (US$ 302.58 per ton and US$ 201.50 per arroba, respectively) and projected soybean and pasture productivity for each of the two deforestation scenarios with and without decreases in yields. Under each scenario, total annual revenues are calculated by weighting soy and pasture revenues using the simulated future area under crops and pastures. (V) We calculated the net present value (NPV, Supplementary equation (14)) of future revenues for SBA using as the discount rate the Selic interest rate of 3.75% (the Selic is determined by Brazil’s Central Bank) and converted NPV into an equivalent annual annuity (EAA, Supplementary equation 15). (VI) The difference between the total NPV of revenues under the WEG versus the SEG scenario is the opportunity cost of the SEG scenario. Similarly, the difference between EAA of revenues under the WEG versus the SEG scenario is the annual opportunity cost of the SEG scenario.

## Supplementary information

Supplementary Information

## Data Availability

All the data that support the findings of this study are obtained from publicly available sources. Annual deforestation data are obtained from the Program to Calculate Deforestation in the Amazon (PRODES) (http://terrabrasilis.dpi.inpe.br/app/map/deforestation?hl=pt-br). Precipitation data use the TRMM (TMPA/3B43) Rainfall Estimate L3 1 month 0.25-degree × 0.25-degree V7 from the U.S. National Aeronautics and Space Administration (https://disc.gsfc.nasa.gov/datasets/TRMM_3B43_7/summary). Elevation data come from the Shuttle Radar Topography Mission (SRTM) from the National Aeronautics and Space Administration of U.S. (https://www.usgs.gov/centers/eros/science/usgs-eros-archive-digital-elevation-shuttle-radar-topography-mission-srtm-non?qt-science_center_objects=0#qt-science_center_objects).

## Code availability

All analytical and simulation models were developed using either Dinamica EGO (dinamicaego.com) or R (r-project.org). EGO models and R codes are available at 10.6084/m9.figshare.14192519.v1.
