# Supplementary material for: Deforestation reduces rainfall and agricultural revenues in the Brazilian Amazon
Source: Nat Commun. 2021 May 10;12:2591. doi: 10.1038/s41467-021-22840-7 (PMC8110785; doi:10.1038/s41467-021-22840-7)
Supplement: Supplementary file 1 — Supplementary Information [file 41467_2021_22840_MOESM1_ESM.pdf]

Supplementary Information

Deforestation reduces rainfall and agricultural revenues in the Brazilian Amazon

Argemiro Teixeira Leite-Filho[1]*, Britaldo Silveira Soares-Filho[1], Juliana Leroy Davis[1], Gabriel Medeiros Abrahão[2], Jan Börner[3]

Affiliations

[1]Centre for Remote Sensing, Federal University of Minas Gerais, Belo Horizonte, Brazil.
[2]Department of Agricultural Engineering, Federal University of Viçosa, Viçosa, Brazil.
[3]Centre for Development Research, University of Bonn, Bonn, Germany.

*Correspondence and requests for materials should be addressed to A.T.L-F. (email: argemiro@csr.ufmg.br).

This file includes:

1. SUPPLEMENTARY METHODS

1.1. Modelling the spatial variability of annual rainfall

1.2. Detrending procedure

1.3. Verification of the detrending method

1.4. Separating the effects of forest loss from the mean long-term trend in rainfall

1.5. Environmental governance scenarios

1.5.1. The weak environmental governance (WEG) scenario

1.5.2. The strong environmental governance (SEG) scenario

1.6. Effects of deforestation on agricultural production and economic analysis

1.6.1. How a reference yield is modified as the local climate changes due to incremental forest loss

1.6.1.1. Change in revenue per hectare for Soybeans

1.6.1.2. Change in revenue per hectare for beef

2. SUPPLEMENTARY RESULTS AND DISCUSSION

2.1. How relevant these rainfall reductions are in magnitude

2.2. Additional Evidence for forest loss causation of rainfall reduction

2.3. Spatially-explicit signal of the reduction of rainfall in the region

3. SUPPLEMENTARY REFERENCES

SUPPLEMENTARY FIGURES:

Supplementary Figure 1. **Mean observed annual rainfall in southern Brazilian Amazon**. Beige to blue gradient colour indicates the total annual rainfall (mm $year^{-1}$) from the Tropical Rainfall Measuring Mission between 1999 and 2019.

Supplementary Figure 2. **Regression models fitted to TRMM rainfall data along transect from NW to SE across Southern Brazilian Amazon**. **a** Linear model. **b** Exponential model, **c** Second-degree polynomial model. **d** Third-degree polynomial model. **e** Potential model and **f** Logarithmic model. All regressions are statistically significant with $p < 0.005$.

Supplementary Figure 3. **Mean annual rainfall estimates for the southern Brazilian Amazon**. Beige to blue gradient color indicates the total annual rainfall (mm $year^{-1)}$ calculated by using a second-degree polynomial model. Note that the map clearly shows the signal of the South Atlantic Convergence Zone.

Supplementary Figure 4. **3-step procedure to calculate the anomalies.** Diagram with method steps to calculate the annual rainfall anomaly for each grid cell. *i, j, t* are the subscripts representing space (*i,j*) and time (*t*) dimensions, $\mathrm{P}_{i,j,t}$ are the annual rainfall values in mm, $\widehat{\mathrm{P}}_{i,j}$ are the estimated values of rainfall due to geographical location and elevation; $\mathrm{P}*_{i,j,t}$ is the difference between observed annual rainfall values and the estimated ones due to geographical location and elevation; $\overline{\mathrm{P}}_{\mathrm{t}}$ are the annual averages of rainfall calculated throughout the study region; $\mathrm{P}'_{i,j,t}$ are the residual annual rainfall anomalies; φ, λ, ζ are the subscripts representing latitude, longitude (in degrees), and elevation (in meters).

Supplementary Figure 5. **Residues between raw values of observed annual rainfall and the estimated values due to geographical location and elevation**. Brown to blue gradient color indicates residues between raw values of observed annual rainfall from the Tropical Rainfall Measuring Mission data and the estimated values after equation (S1).

Supplementary Figure 6. **Mean annual rainfall anomalies and the best-fit linear regression with ENSO.** Mean annual rainfall anomalies between 1999 and 2019 and the best-fit linear regression (red dashed line) with El Nino Southern Oscillation (ENSO) indexes in the Niño 3.4 region.

Supplementary Figure 7: **Standard deviation of annual rainfall.** Brown to blue gradient color indicates standard deviation of annual rainfall within each 28x28-km grid cell in the period of 1999 to 2019.

Supplementary Figure 8. Estimated rainfall reduction by 2019 due to forest loss in grid cells that have crossed the critical threshold of forest loss.

Supplementary Figure 9. **Simulated agricultural land use by 2050.** Simulated agricultural land use by 2050 for **a** SEG, **b** and WEG. Deforested area (yellow) by 2019 according to Program to Calculate Deforestation in the Amazon (PRODES).

Supplementary Figure 10. **Spatial association between forest loss and estimated anomalies for 2019.** Spatial variability in anomalies and the Cramer's V[10] and the

Spearman Rank Order Correlation coefficient to measure the spatial association between forest loss and estimated anomalies for **c** and **d** cells that have reached the critical threshold of forest loss and **a** and **b** cells that have not crossed this limit. Brown to blue gradient color indicates precipitation anomalies (mm) for 2019. Red to pink gradient color indicates forest loss (%) within 28 km grid cells. Each point represents the location of a State Capital.

Supplementary Figure 11. **Significant effect of Maximum cumulative water deficit on deforestation.** One degree grid cells with a significant (α = 0.05) temporal effect of the Maximum Climatological Water Deficit on deforestation (MCWD; % yr^{-1} mm^{-1}). Colors indicate change in deforestation for each mm reduction in MCWD as a percentage of the average annual local forest suppression. Note that cells with statically significant effect concentrate in the highly humid forests of Northwestern Amazon.

Supplementary Figure 12. Difference of mean annual rainfalls between two periods: 1999-2009 and 2010-2019.

Supplementary Figure 13. **Length of the dry season in Southern Amazon**. Length of the dry season in Southern Amazon between 1980 and 2010 obtained from National Oceanic and Atmospheric Administration/US (NOAA) Climate Diagnostics Center (CDC) daily precipitation gridded data product, version SA24 data. Years are classified into Long dry season (LDS) and short dry season (SDS) using a 0.75σ threshold.

SUPPLEMENTARY TABLES:

Supplementary Table 1: Regression models fitted to TRMM rainfall data in Southern Brazilian Amazon.

Supplementary Table 2: Residues from the second-degree polynomial model

Supplementary Table 3: Coefficients of the second-degree polynomial model

Supplementary Table 4: Spearman Rank-Order correlation coefficients between rainfall values predicted for the entire time-period of study and those predicted using equations derived for three specific time-periods.

Supplementary Table 5: Mean annual anomalies for study region between 1999 and 2019.

Supplementary Table 6: Cell grid groups divided by forest loss fraction for the periods 1980–1996 and 1997–2012.

Supplementary Table 7: Two-Sample t-test with unequal sample sizes and unequal variance of means of annual rainfall anomalies ($P_{i,j,t}$) for cell groups 1 and 2 for the 1999–2009 and 2010–2019 periods.

1. SUPPLEMENTARY METHODS

1.1. Modelling the spatial variability of annual rainfall:

The TRMM data show a swath of high values of annual rainfall extending from NW to SE (Figure S1), which may relate to the South Atlantic Convergence Zone (SACZ).

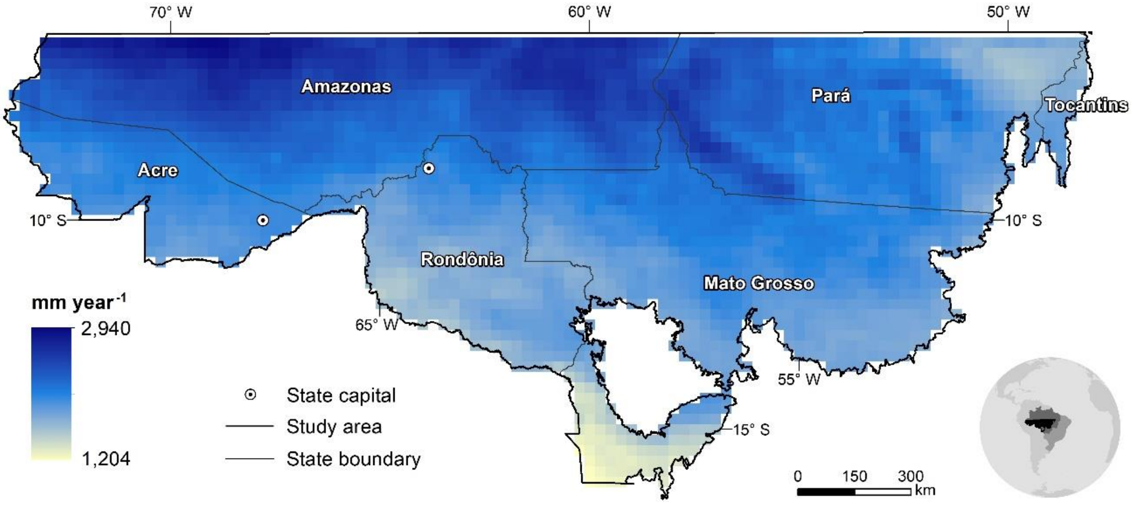

Supplementary Figure 1. **Mean observed annual rainfall in southern Brazilian Amazon**. Beige to blue gradient colour indicates the total annual rainfall (mm year^{-1}) from the Tropical Rainfall Measuring Mission between 1999 and 2019.

We computed a rainfall profile along a transect from NW to SE to evaluate this gradient by testing different non-linear regression models to select the one that provides a good fit to the data (Supplementary Figure 2). Although the third-degree polynomial model yielded a slightly higher fitting, the gains are not so expressive when compared with the results of a second-degree polynomial model. To avoid overfitting common to high degree polynomial models, we opted for the second-degree polynomial model.

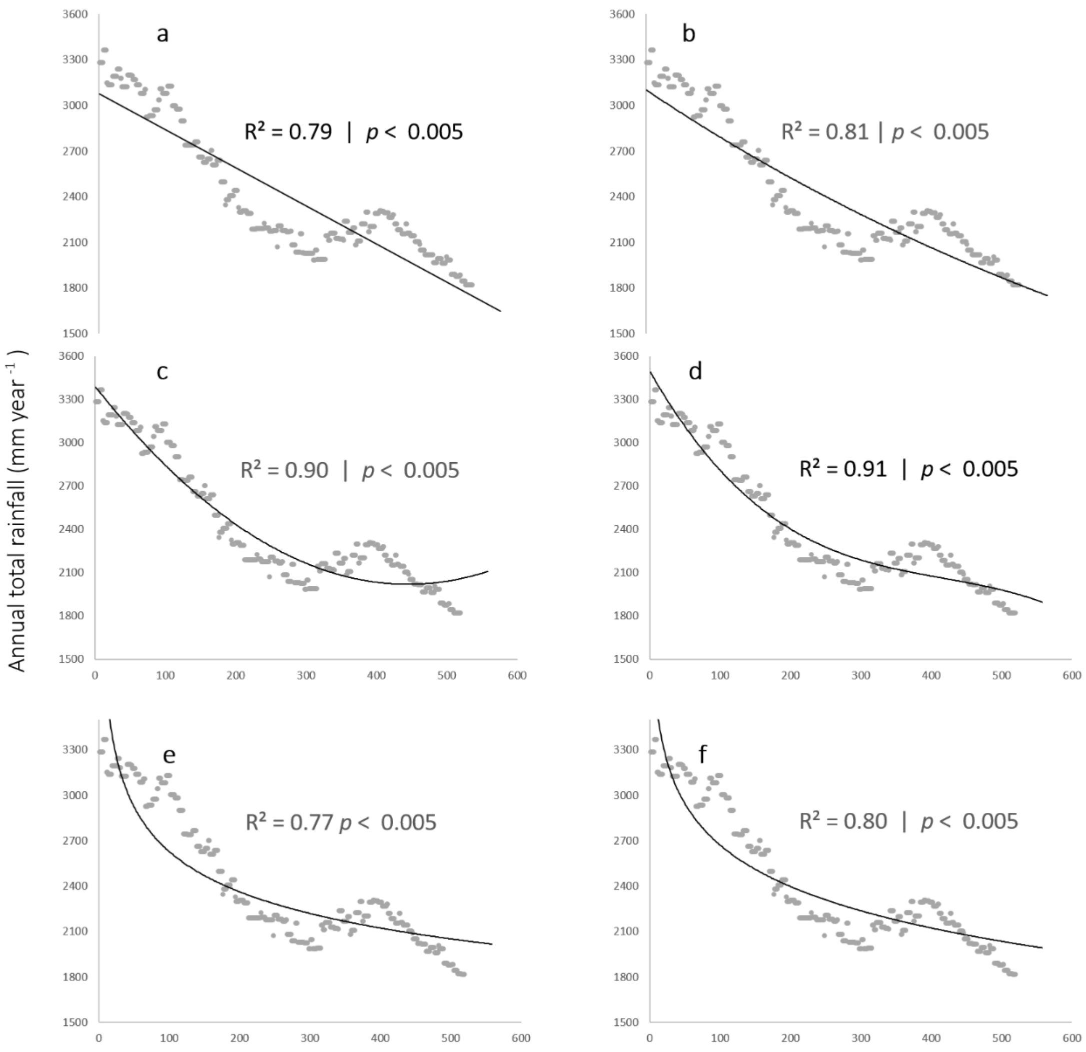

Supplementary Figure 2. **Regression models fitted to TRMM rainfall data along transect from NW to SE across Southern Brazilian Amazon**. **a** Linear model. **b** Exponential model, **c** Second-degree polynomial model. **d** Third-degree polynomial model. **e** Potential model and **f** Logarithmic model. All regressions are statistically significant with $p < 0.005$.

We also applied these non-linear regression models to all cells of the study region and analysed the distance between known data points and the ones predicted with the regression models (the residual standard error; Supplementary Table 1).

Supplementary Table 1: Regression models fitted to TRMM rainfall data in Southern Brazilian Amazon.

Model	R^2	*p*-value	Residual standard error
Third-degree polynomial	0.75	< 0.005	193.6 mm
Second-degree polynomial	0.72	< 0.005	204.6 mm
Logarithmic	0.67	< 0.005	222.9 mm
Exponential	0.67	< 0.005	223.5 mm
Linear	0.66	< 0.005	226.2 mm
Potential	0.65	< 0.005	228.1 mm

Supplementary Figure 3 shows the mean annual rainfall estimates (1999 to 2019) calculated by using a second-degree polynomial model. Note that the map clearly depicts the signal of the SACZ with a good agreement with the observed values (r^2 = 0.72; p < 0.005). Table S2 shows the model residues and Table S3 shows the summary statistics for the second-degree polynomial model.

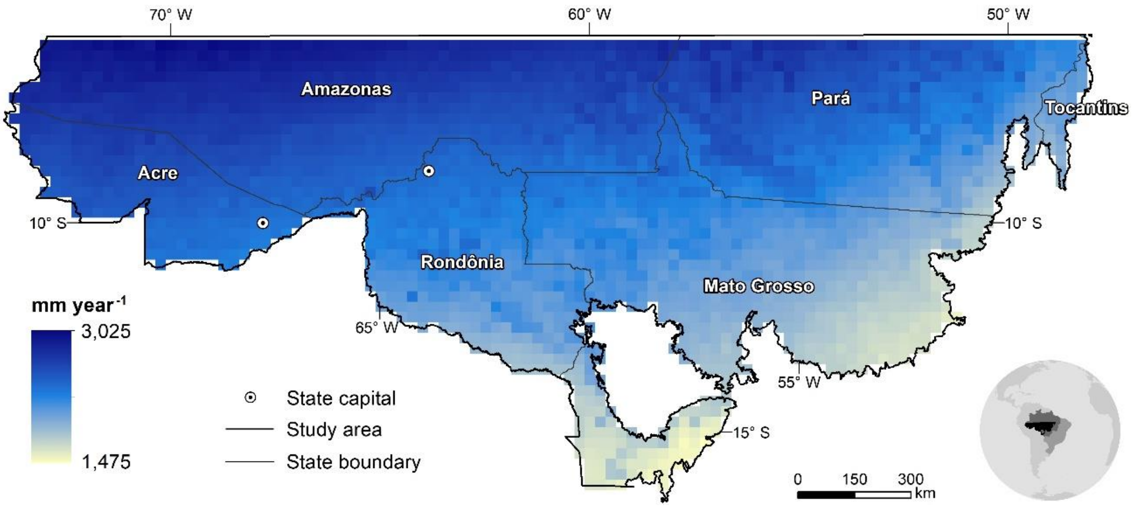

Supplementary Figure 3. **Mean annual rainfall estimates for the southern Brazilian Amazon**. Beige to blue gradient color indicates the total annual rainfall (mm year^{-1}) calculated by using a second-degree polynomial model. Note that the map clearly shows the signal of the South Atlantic Convergence Zone.

Supplementary Table 2: Residues from the second-degree polynomial model

Min	1Q	Median	3Q	Max
-842.32	-148.09	2.44	160.97	671.94

Supplementary Table 3: Coefficients of the second-degree polynomial model

	Estimate	Standard Error	t_{value}	Pr(>|t|)
Intercept	$-5.29x10^{3}$	$4.92\ x10^{2}$	-10.74	< 0.001
latitude	$2.30x10^{2}$	$1.37x10^{1}$	16.74	< 0.001
atitude²	4.69	6.68E-01	7.019	< 0.001
longitude	$-2.71x10^{2}$	$1.61x10^{1}$	-16.813	< 0.001
longitude²	-1.99	$1.31x10^{-1}$	-15.221	< 0.001
altitude	1.41	$1.89\ x10^{-1}$	7.442	< 0.001
altitude²	$-1.02x10^{-3}$	$3.15\ x10^{-4}$	-3.247	< 0.001

We tested the assumption that these coefficients are constant over time by randomizing the annual data of the TRMM. We then calculated a correlation coefficient, i.e. Spearman Rank-Order Correlation Coefficients, between predicted values for the entire period of study and predicted values for three different time-periods. We concluded that, although there are small differences between the equations' coefficients, the results from the equation for the entire period of analysis strongly correlates[1] with the ones for the three-separate time-periods (Supplementary Table 4). As a result, we find that Interannual changes in the spatial patterns are non-monotonic (as would be expected due to e.g. ENSO), and as such, should not bias our estimated effects.

Supplementary Table 4: Spearman Rank-Order correlation coefficients between rainfall values predicted for the entire time-period of study and those predicted using equations derived for three specific time-periods.

Period	Spearman's rho	Interpretation
1999 to 2006	0.58	Strong relationship
2007 to 2013	0.75	Very Strong relationship
2014 to 2019	0.56	Strong relationship

1.2. Detrending procedure

To minimize omitted variable bias in our regression analysis, we firstly removed the effects of factors other than deforestation that may affect rainfall across both time and space. To remove the trends associated with geographic location and elevation, as well as part of the interannual variability associated with large-scale climate mechanisms, hence isolating the deforestation effects from those of natural variability, we calculated anomalies of annual rainfall ($P'_{i,j,t}$) using a 3-step procedure, summarized in Supplementary Equations 1, 2 and 3 and Supplementary Figure 4. Notations are as follows:

i, j, t = subscripts representing space (*i,j*) and time (*t*) dimensions.

$\mathrm{P}_{i,j,t}$ = annual rainfall values in mm year^{-1}.

$\widehat{\mathrm{P}}_{i,j}$ = estimated values of rainfall due to geographical location and elevation.

$\mathrm{P}*_{i,j,t}$ = difference between observed annual rainfall values and the estimated ones due to geographical location and elevation.

$\overline{\mathrm{P}}_{t}$ = annual averages of rainfall calculated throughout the study region.

$\mathrm{P}'_{i,j,t}$ = residual annual rainfall anomalies in mm year^{-1}.

φ, λ, ζ = latitude, longitude (in degrees), and elevation (m), latitudes south of the Equator receive negative values, and longitudes west of the Greenwich Meridian receive negative values.

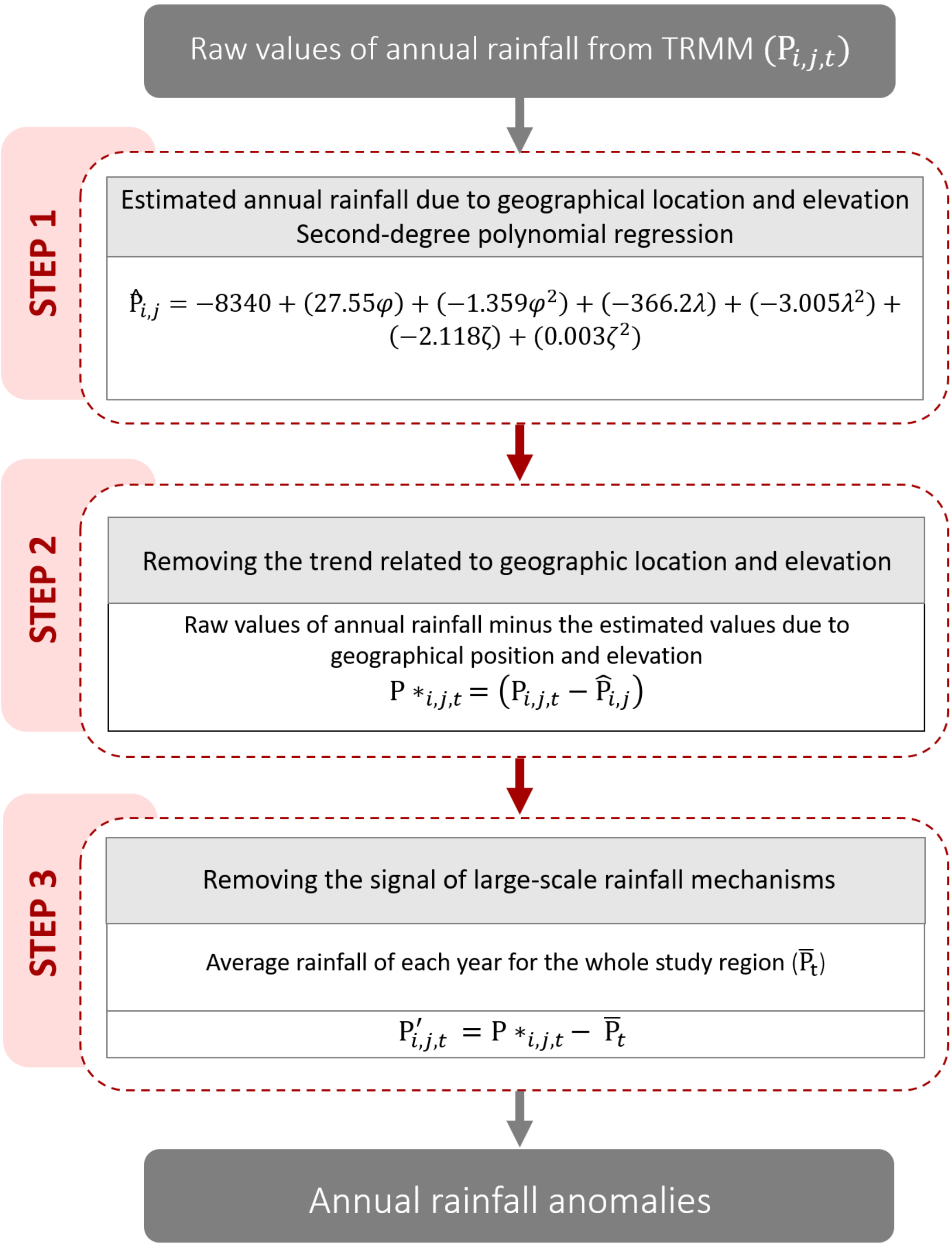

Supplementary Figure 4. **3-step procedure to calculate the anomalies.** Diagram with method steps to calculate the annual rainfall anomaly for each grid cell. *i, j, t* are the subscripts representing space (*i,j*) and time (*t*) dimensions, $P_{i,j,t}$ are the annual rainfall values in mm, $\hat{P}_{i,j}$ are the estimated values of rainfall due to geographical location and elevation; $P*_{i,j,t}$ is the difference between observed annual rainfall values and the estimated ones due to geographical location and elevation; $\overline{P}_t$ are the annual averages of rainfall calculated throughout the study region; $P'_{i,j,t}$ are the residual annual rainfall anomalies; φ, λ, ζ are the subscripts representing latitude, longitude (in degrees), and elevation (in meters).

Step 1.

As aforementioned, the spatial pattern of annual rainfall depicts a swath of high values extending from NW to SE that may be associated with the South Atlantic Convergence Zone. This swath of high rainfall values traverses our study region. To remove this large-scale geographical pattern, we used increasingly complex regression models on latitude, longitude and elevation calculated using the climatological averages from 1999 to 2019. We found that a second-degree polynomial model on latitude, longitude and elevation is sufficient to describe this geographical climatological pattern (equation (S1); $r^2 = 0.72$; $p > 10^{-5}$). Using this model, we computed estimated values of rainfall due to this geographical pattern so that:

$$\hat{P}_{i.j} = -8340 + (27.55\varphi) + (-1.359\varphi^2) + (-366.2\lambda) + (-3.005\lambda^2) + (-2.118\zeta) + (0.003\zeta^2) \quad \text{(S1)}$$

Step 2:

Next, we spatially detrended the values of annual rainfall (i.e., to remove the climatological trend related to geographic location and elevation). To do this, we calculated the difference between raw values of observed rainfall in each year and the estimated values due to geographical position obtained from equation (S1), so that.

$$P*_{i.j.t} = \left(P_{i.j.t} - \widehat{P}_{i.j}\right) \quad \text{(S2)}$$

Since the coefficients of equation (S1) are static over time, the results from equation (S2) represent the observed deviation (anomaly) from the climatological pattern due to the interannual variability.

Large-scale interannual climate phenomena may also affect the annual averages of rainfall values. El Niño Southern Oscillation (ENSO) is the most important interannual variability modulating rainfall in the region, which indeed shows a high correlation with rainfall in our study region (section S1.3; Supplementary Figure 6). Since our time-series is relatively short, there is a bias (albeit weak) toward higher Nino 3.4 indexes, possibly associated with a partial cycle of the Pacific Decadal Oscillation (PDO). As forest losses across the study region increase monotonically as a function of time, Nino 3.4 trend coinciding with forest loss may bias the analysis.

Step 3:

To remove the signal of large-scale factors, such as that of the ENSO, we subtracted the mean annual rainfall for the whole study region from the outputs of equation (S2):

$$\mathrm{P}'_{I,j,t} = \mathrm{P} *_{i.j.t} - \overline{\mathrm{P}}_t \qquad \text{(S3)}$$

The residual is assumed to be an 'anomaly' that is not explained by the geographic location, elevation or large-scale time-varying factors.

1.3. Verification of the detrending method

To show that the detrending method works, we present a map of difference (residues) between raw values of observed annual rainfall and the estimated values due to geographical location and elevation (Supplementary Figure 5). In general, the difference is between 120 mm and -120 mm, i.e., less than 10% of the annual rainfall.

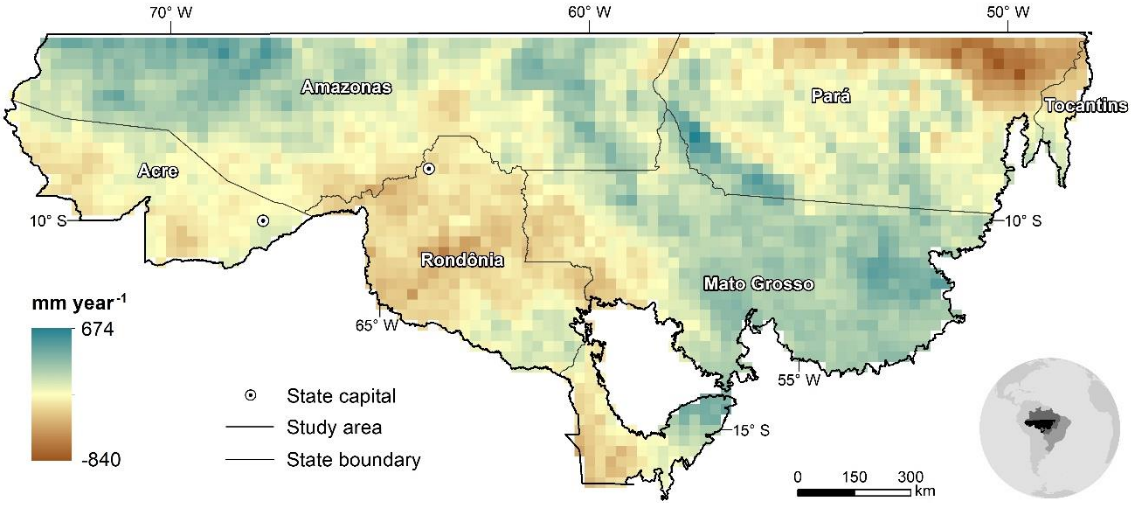

Supplementary Figure 5. **Residues between raw values of observed annual rainfall and the estimated values due to geographical location and elevation**. Brown to blue gradient color indicates residues between raw values of observed annual rainfall from the Tropical Rainfall Measuring Mission data and the estimated values after equation (S1).

As mentioned earlier, we attempted to remove the spatial patterns that might be correlated with deforestation and would thus lead to bias in our regression analyses. We added the step 3 in which we subtracted the annual regional average rainfall from equation (2) to control for possible large-scale mechanisms that affect, on annually basis, the region as whole. This average is highly correlated with the Niño 3.4 Sea Surface Temperature (SST) ENSO index from the US National Oceanic and Atmospheric Administration in January, February and March (JFM - The rainiest months in the Amazon; Supplementary Figure 6).

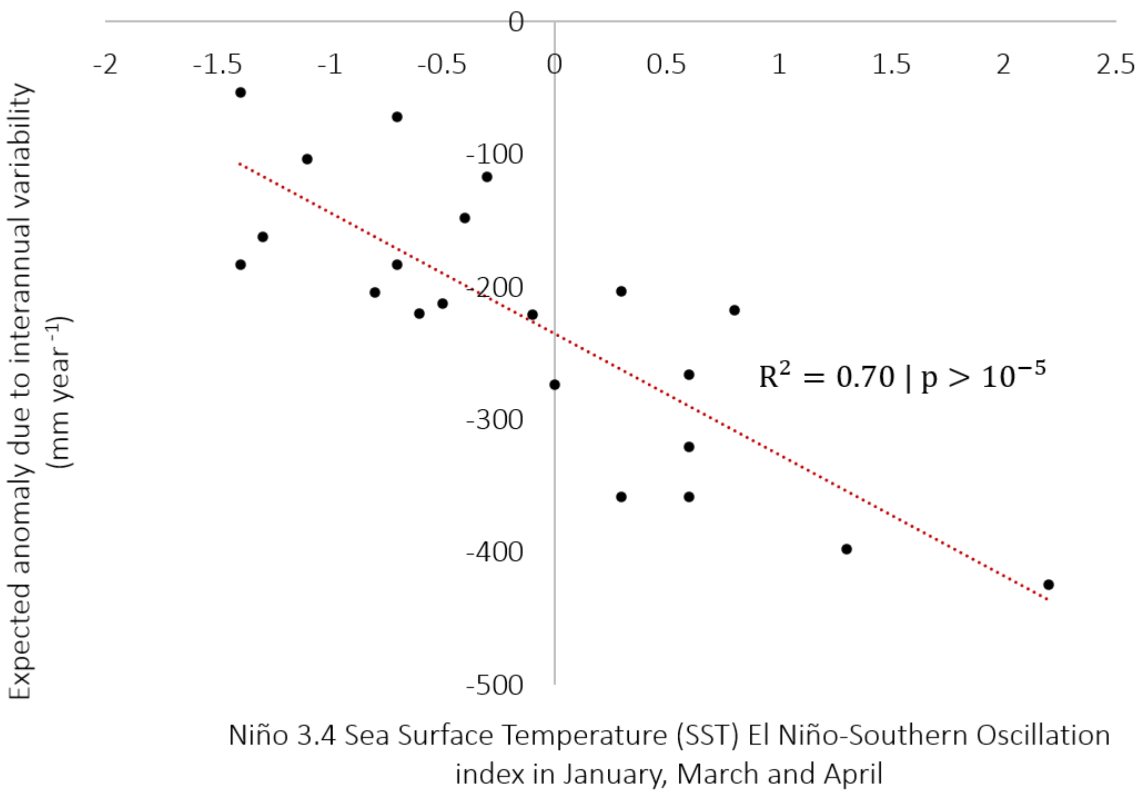

Supplementary Figure 6. **Mean annual rainfall anomalies and the best-fit linear regression with ENSO.** Mean annual rainfall anomalies between 1999 and 2019 and the best-fit linear regression (red dashed line) with El Nino Southern Oscillation (ENSO) indexes in the Niño 3.4 region.

After this step, the residual is assumed to be a "rainfall anomaly" that is not explained by the geographic location, elevation and the interannual variability. As expected, after this procedure, the annual means situate around zero (Table S5).

Supplementary Table 5: Mean annual anomalies for study region between 1999 and 2019.

Year	Mean anomaly
1999	-6.1×10^{-13}
2000	1.12×10^{-13}
2001	-1.3×10^{-13}
2002	1.61×10^{-14}
2003	-7.2×10^{-13}
2004	-2×10^{-13}
2005	-3.2×10^{-13}
2006	-3.4×10^{-13}

2007	8.9 x 10^{-14}
2008	-2.8 x 10^{-14}
2009	-1.1 x 10^{-13}
2010	9.97 x 10^{-13}
2011	8.85 x 10^{-14}
2012	-9.6E x 10^{-14}
2013	1.01 x 10^{-13}
2014	-3.1 x 10^{-13}
2015	4.39 x 10^{-13}
2016	-1.1 x 10^{-13}
2017	-3.3 x 10^{-14}
2018	8.97 x 10^{-14}
2019	-1.9 x 10^{-13}

1.4. Separating the effects of forest loss from the mean long-term trend in rainfall

Since historical advance of the deforestation frontier (from south to north and east to west) could have possibly coincided with climate trends, we separated the effects of forest loss from the mean long-term trend in rainfall by performing a t-test. The t-test compares the annual rainfall between cells with smaller forest loss and cells that experienced larger forest loss within two separated periods: 1999–2009 and 2010–2019 (Tables S6 and S7).

Supplementary Table 6: Cell grid groups divided by forest loss fraction for the periods 1980–1996 and 1997–2012.

Group	Mean Latitude	Mean Longitude	Mean elevation	Period	Mean deforestation (%)
1	-8.1034	-54.2299	178	1999–2009	25.1
				2010–2019	27.3
2	-9.0378	-55.9949	191	1999–2009	24.9
				2010–2019	57.2

Results demonstrate that the cells with smaller forest loss over the periods (not reaching 55-60% forest losses) have an annual rainfall increase of +96.9±12.65 mm, whereas cells with larger forest losses (exceeding 55-60% forest loss) have an annual decrease of -306.4±42.77 mm (Table S7). Both relations are significant at $p = 10^{-5}$.

Supplementary Table 7: Two-Sample t-test with unequal sample sizes and unequal variance of means of annual rainfall anomalies ($P_{i,j,t}$) for cell groups 1 and 2 for the 1999–2009 and 2010–2019 periods.

Group	µ 1999–2009	µ 2010–2019	S^2 1999–2009	S^2 2010–2019	t_{calc}	$P_{1\text{-tailed}}$
1	1998.4 mm	2095.3 mm	120.12 mm	131.35 mm	0.252	0.249
2	2079.3 mm	1772.9 mm	129.11 mm	170.56 mm	-1.983	0.012

1.5. Environmental governance scenarios

1.5.1. The weak environmental governance (WEG) scenario

The weak environmental governance scenario assumes the abandonment of current deforestation control policies, as well as strong political support for predatory agricultural practices. In practice, the environmental achievements attained in the Amazon since 2005 would be completed undone by 2025. This represents the worst-case scenario and should be viewed as a complete deconstruction of environmental governance in Brazil with severe impacts on deforestation rates, which could potentially return to pre-2005 levels. As a result, annual deforestation rates would reach 27,000 km^2 by 2025.

1.5.2. The strong environmental governance (SEG) scenario

The strong environmental governance (SEG) scenario assumes the expansion of current deforestation command-and-control policies under full political support for the country's environmental agenda, including the through implementation of the Forest Code

alongside economic incentives for forest conservation. Annual deforestation in the Amazon would be reduced from 7,989 and 9,483 km^2 in 2016 and 2015, respectively, to 4,000 km^2 by 2030.

1.6. Effects of deforestation on agricultural production and economic analysis

The economic analysis is based on simulations of deforestation under a weak (WEG) and strong (SEG) environmental governance scenario[2] described earlier. These associated economic losses are estimated using an origin-destination rainfall relationship at a broader scale[3]. These analyses complement the manuscript aimed at illustrating how important deforestation-rainfall effects can be to the region's economy.

1.6.1. How a reference yield is modified as the local climate changes due to incremental forest loss

Strand et al.[3] ran computer simulations using the Integrated Model of Land Surface Processes (INLAND)[4] to calculate how a reference yield is modified as the local climate changes due to incremental forest loss. The soybean yield and pasture productivity calculated using the INLAND model is a function of a set of climate variables, crop and soil parameters. Strand et al. used the climate variables output by the Community Climate Model version 3, coupled to the Integrated Biosphere Simulator version 2.6.4 (named CCM3-IBIS) for modelling the impact of incremental deforestation scenarios on rainfall regulation. In these simulations, Strand et al. used the climate variables output from the climate model for each deforestation scenario F*x*, where *x* is the percentage of total Amazon forest loss. The 10% Amazon deforestation scenario (F_{10}) is the baseline for calculations of relative differences, as follows:

1.6.1.1. Change in revenue per hectare for Soybeans:

The change in soybean yield in each deforestation scenario is denoted below:

$\Delta \mathrm{Y}_{i,j}^{d} = (\mathrm{Y}_{i,j}^{\mathrm{F}x} - \mathrm{Y}_{i,j}^{\mathrm{F10}})$ if d = F*x*, *x* = 10, 20, …, 40. (S4)

where *i* and *j* refer to latitude and longitude, respectively; $\Delta \mathrm{Y}_{i,j}^{d}$ is the change in soybean yield in cell *i,j* for a deforestation scenario d (ton ha^{-1}); $\mathrm{Y}_{i,j}^{\mathrm{F}x}$ is the soybean yield (ton ha^{-1}) in cell *i,j* in one of the deforestation scenarios Fx (x = 10, 20, …, 40% of Amazon deforestation); $\mathrm{Y}_{i,j}^{F10}$ is the soybean yield in cell *i,j* in the standard scenario F_{10} (ton ha^{-1}); and A^{Fx} is the total area deforested in scenario F*x* (km^2). If the deforestation scenario *d* is different than one of the standard scenarios F*x* (*x* = 10, 20, …,40% of Amazon deforestation), then the result is interpolated using equation (S5):

$\Delta \mathrm{Y}_{i,j}^{d} = \sum_{x=20}^{40} \left(\mathrm{Y}_{i,j}^{\mathrm{F}x} - \mathrm{Y}_{i,j}^{\mathrm{F10}}\right) . \left(\frac{\mathrm{A}_{d}^{\mathrm{F}x}}{\mathrm{A}^{\mathrm{F}x}}\right)$ (S5)

The percentage change ΔYij % in soybean yield is as follows:

$\Delta \mathrm{Yij}\ \% = \frac{\Delta Y_{i,j}^{d}}{Y_{i,j}^{F10}} . 100$ (S6)

Change in revenue per hectare is calculated by multiplying the simulated yield and the price of a ton of soybean:

$\Delta \mathrm{R}_{ij}^{ha} = \frac{\Delta \mathrm{Y}ij\ \%}{100} . Y_{i,j}^{\mathrm{ref}} . \mathrm{P}_{\mathrm{soy}}$ (S7)

Where $\Delta \mathrm{R}_{ij}^{ha}$ is the change in soybean revenue per hectare in cell *i,j* (US$ ha^{-1}) and P_{soy} is the price of a ton of soybean (US$ ton^{-1}).

1.6.1.2. Change in revenue per hectare for cattle beef

The cattle beef production is derived using the regional stocking rates as of 2012:

$$\mathrm{C}_{ij}^{ref} = \frac{\mathrm{S}_{ij}^{\mathrm{ref}}.\mathrm{W}.\mathrm{r}}{a.\mathrm{t_s}} \quad \text{(S8)}$$

where $\mathrm{C}_{ij}^{\mathrm{ref}}$ is the reference cattle beef production (arroba/ha/year). $\mathrm{S}_{ij}^{\mathrm{ref}}$ is the reference stocking rate as of 2012 (heads ha^{-1}); W is the national average weight per animal (540 kg $head^{-1}$); *r* is the average ratio dead weight/live weight (*r* = 0.41); *a* is the conversion factor from arroba to kg (a = 15kg $arroba^{-1}$); and t*s* is the average animal age at slaughter (ts = 2 years).

Change in pasture productivity P in each deforestation scenario is calculated using equations (S9) and (S10):

$$\Delta\mathrm{P}_{i,j}^{d} = (\mathrm{P}_{i,j}^{\mathrm{F}x} - P_{i,j}^{\mathrm{F10}}) \text{ if d = F}x,\ x = 10, 20, ..., 40. \quad \text{(S9)}$$

where *i* and *j* refer to latitude and longitude, respectively; $\Delta\mathrm{P}$ is the change in pasture productivity (ton/ha) in cell *i,j* in one of the deforestation scenarios Fx (x = 10, 20, ..., 40% of Amazon deforestation) *d*; P is the pasture productivity (Kg-C.m^{-2}.yr^{-1} ha^{-1}) in cell *i,j* in one of the deforestation scenarios F*x* (x = 10, 20, ..., 40% of Amazon deforestation); $\mathrm{P}_{i,j}^{\mathrm{F10}}$ is the pasture productivity in pixel *i,j* in the standard scenario F_{10} (ton ha^{-1}); and $\mathrm{A}^{\mathrm{F}x}$ is the total area deforested in scenario F*x* (km^2). If the generic deforestation scenario d is different than one of the standard scenarios F*x* (*x* = 10, 20, ..., 40), then the result is interpolated using equation (S10):

$$\Delta\mathrm{P}_{i,j}^{d} = \sum_{x=20}^{40}\left(\mathrm{P}_{i,j}^{\mathrm{F}x} - \mathrm{P}_{i,j}^{\mathrm{F10}}\right).\left(\frac{\mathrm{A}_{d}^{\mathrm{F}x}}{\mathrm{A}^{\mathrm{F}x}}\right) \quad \text{(S10)}$$

The percentage change $\Delta\mathrm{P}ij$ % in pasture productivity is denoted as follows:

$$\Delta\mathrm{P}ij\,\% = \frac{\Delta\mathrm{P}_{i,j}^{d}}{\mathrm{P}_{i,j}^{\mathrm{F10}}}.100 \quad \text{(S11)}$$

Equation (S12) then calculates the change in cattle beef production:

$$C_{ij}^{d} = \frac{C_{ij}^{\text{ref}}.\ \Delta \text{P}ij\ \%}{100} \qquad \text{(S12)}$$

The revenue per hectare is calculated by multiplying the cattle beef production per hectare per year and the price of the arroba:

$$\text{E}_{ij}^{ha} = \text{C}_{i,j}^{d}.\,\text{P}_{\text{arroba}} \qquad \text{(S13)}$$

where E_{ij}^{ha} is the cattle beef revenue per hectare per year in cell *i,j* (US\$ ha^{-1} yr^{-1}) and $\text{P}_{\text{arroba}}$is the price of an arroba of dead weight (US\$ $arroba^{-1}$).

Next, we projected productivity change until 2050 from estimates of soybean and pasture productivity losses due to lower rainfall caused by Amazon region-wide deforestation under WEG and SEG scenarios. To do so, we adjusted soybean productivity projections (3.7 ton $hectare^{-1}$)[5] and pasture productivity projections (2.9 arroba $hectare^{-1}$)[6] by the average productivity losses. We computed annual revenues in US$ per hectare using current soybean and cattle arroba prices (US$ 302.58 per ton and US$ 201.50 per arroba, respectively) and projected soybean and pasture productivity for each of the two forest loss scenarios (WEG and SEG) with and without decreases in yields. Under WEG and SEG scenarios, total annual revenues are calculated by weighting soy and pasture revenues using the simulated future croplands and pasturelands. Next, we calculate the Net Present Value (NPV; Eq. S14) of future revenues for the Southern Amazon using as discount rate the Special System of Clearance and Custody rate (Selic interest rate) of 3.75% (the Selic interest rate is determined by Brazil's Central Bank).

$$\text{NPV} = \frac{\text{R}_t}{(1+\text{i})^{\text{t}}} \qquad \text{(S14)}$$

Where t is the time of the cash flow, i is the Selic interest rate and R_t is the net cash flow i.e. cash inflow – cash outflow, at time t. We computed the difference between the total NPV of revenues under the WEG versus the SEG scenario is the opportunity cost of the SEG scenario.

Finally, we converted NPV into Equivalent Annual Annuity (EAA; Eq. 15).

$$\text{EAA} = \frac{i\ x\ \text{NPV}}{1-(1+i)^{-\text{N}}} \qquad \text{(S15)}$$

where i is the Selic discount rate, NPV is the net present value calculate trough equation (S14) and N is the simulation period (i.e., the planning horizon, in years). Similarly, the difference between total EAA of revenues under the WEG versus the SEG scenario is the annually opportunity cost of the SEG scenario.

2. SUPPLEMENTARY RESULTS AND DISCUSSION

2.1. How relevant these rainfall reductions are in magnitude

Our estimates of 2096 mm year^{-1} for the study region using the TRMM database have a standard deviation (σ) of 245.2 mm year^{-1} (≈11%). Considering our results (-4.9 mm of rainfall reduction per each 1% increase in forest loss), in a hypothetical case of 80% of forest loss (in a 28-km cell), the tantamount reduction would be of ≈ 19%, thus 8% larger than the typical year-to-year variability. However, it is important to stress that these inferences are for the study region as a whole. Particular regions may be more or less affected according to their locations. As presented earlier, there is a striking spatial variability in the standard deviation of annual rainfall (Supplementary Figure 7).

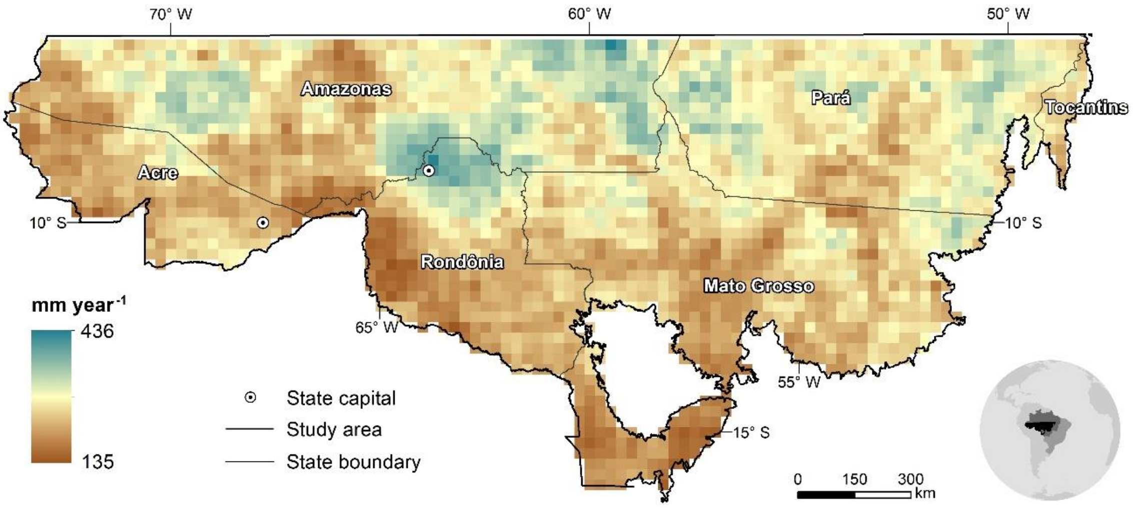

Supplementary Figure 7: **Standard deviation of annual rainfall.** Brown to blue gradient color indicates standard deviation of annual rainfall within each 28x28-km grid cell in the period of 1999 to 2019.

The smallest interannual variability is verified in grid cells that are located in south and southwest of the Amazon, in the same regions where the annual rainfall is lower; consequently, in those regions rainfall reduction due to deforestation is proportionally larger. For example, in some regions rainfall reduction by 2019 may have already reached

48% of the annual rainfall (Supplementary Figure 8). This has a large implication for agriculture since those areas mostly affected by rainfall reduction are the ones where soy croplands concentrate or are likely to expand by 2050 (Supplementary Figure 9).

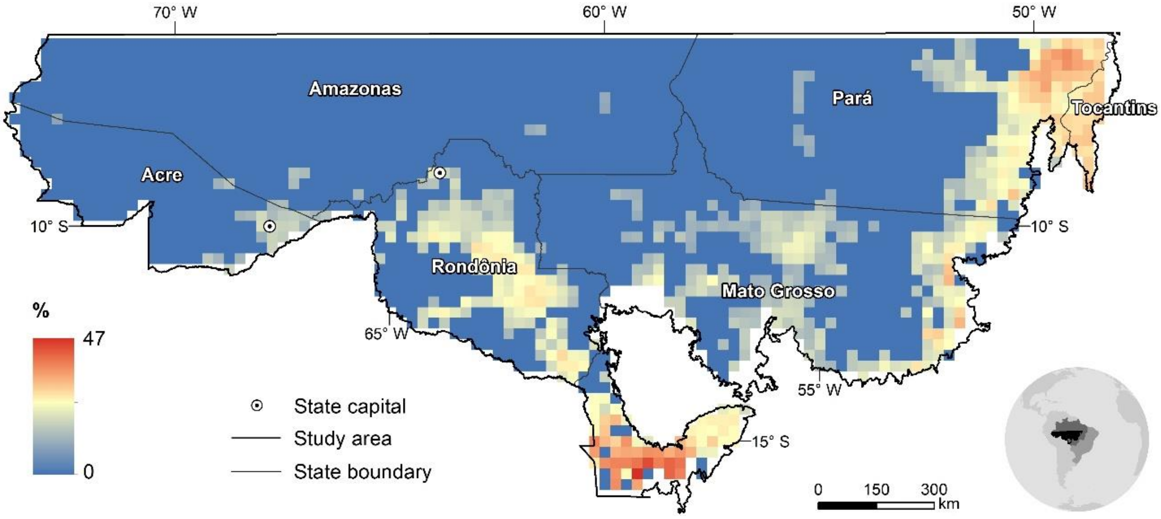

Supplementary Figure 8. Estimated rainfall reduction by 2019 due to forest loss in grid cells that have crossed the critical threshold of forest loss.

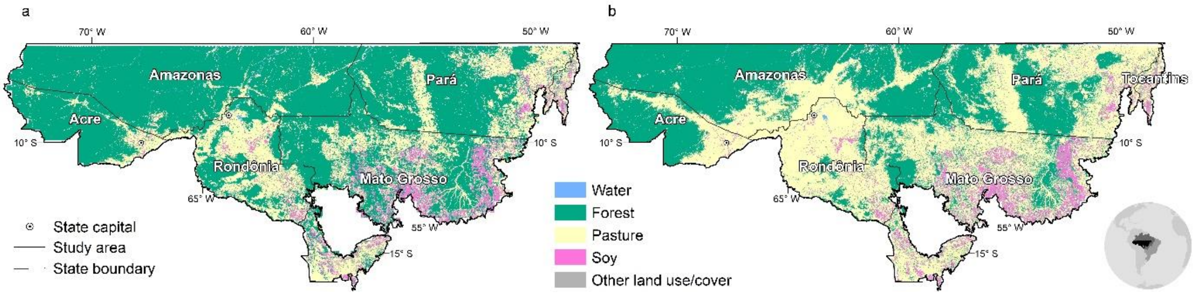

Supplementary Figure 9. **Simulated agricultural land use by 2050.** Simulated agricultural land use by 2050 for **a** SEG, **b** and WEG. Deforested area (yellow) by 2019 according to Program to Calculate Deforestation in the Amazon (PRODES).

The fact that the reduction in rainfall could be smaller than the typical year-to-year variability in some of the grid cells does not mean that there is no impact on agricultural production. Since we have isolated the signal of deforestation, the effect of deforestation on rainfall is additional to the interannual variability, further increasing risks of crop failure especially in dry or drought years. Furthermore, crop yields and the adoption of double cropping systems vary significantly from year to year in the region, partially

because of the interannual climate variability[8,9]. Therefore, it is reasonable to assume that even changes that are smaller than the interannual variability may be relevant to agriculture in the region.

2.2. Evidence for forest loss causation of rainfall reduction

We also evaluated the spatial variability in anomalies calculating the Cramer's V[10] and the Spearman Rank Order Correlation coefficient[11] to measure the spatial association between forest loss and estimated anomalies for cells that have reached the critical threshold of forest loss and cells that have not crossed this limit. As expected, after isolating the deforestation effects, anomalies exhibit a spatial variability correlated only with forest loss (Supplementary Figure 10), thus an additional evidence for forest loss causation of rainfall reduction.

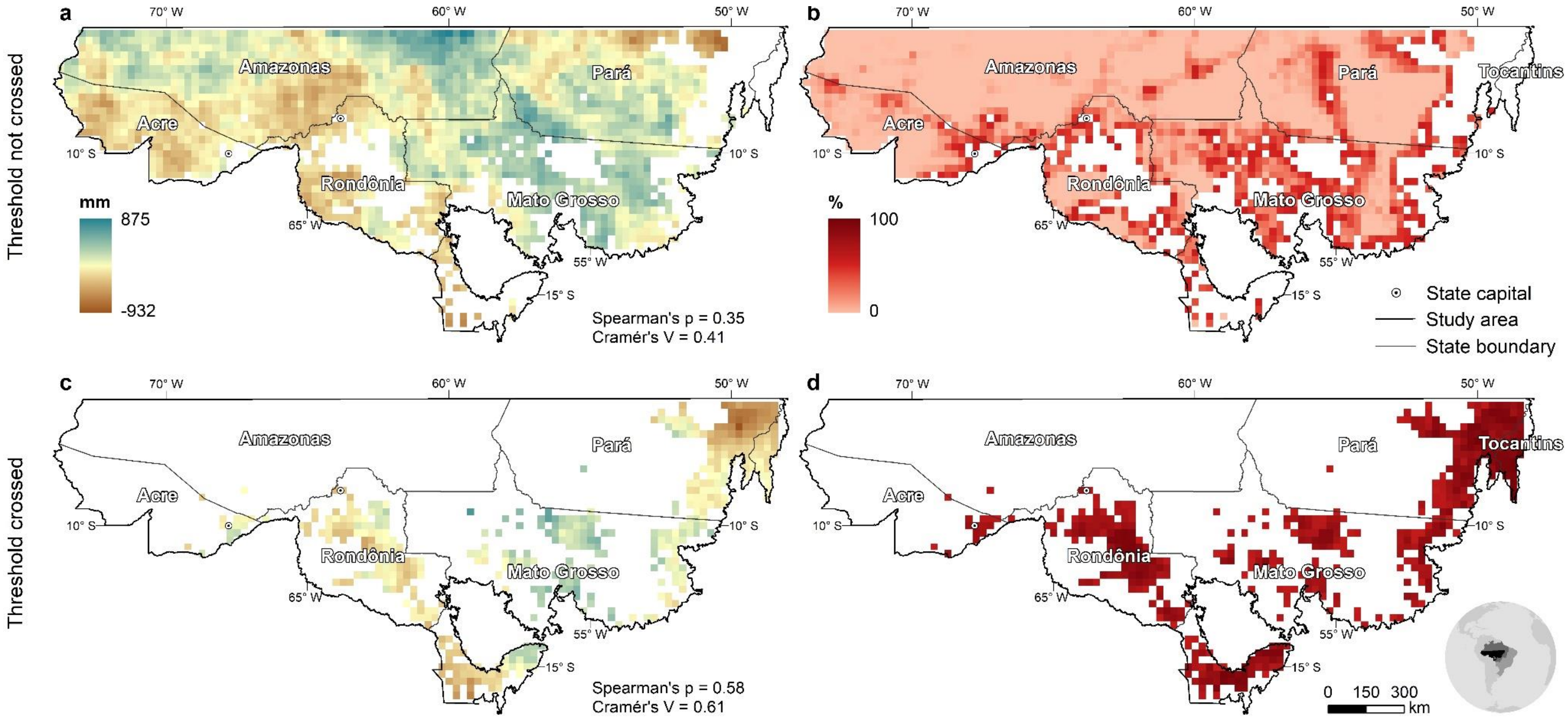

Supplementary Figure 10. **Spatial association between forest loss and estimated anomalies for 2019.** Spatial variability in anomalies and the Cramer's V[10] and the Spearman Rank Order Correlation coefficient to measure the spatial association between forest loss and estimated anomalies for **c** and **d** cells that have reached the critical threshold of forest loss and **a** and **b** cells that have not crossed this limit. Brown to blue gradient color indicates precipitation anomalies (mm) for 2019. Red to pink gradient color indicates forest loss (%) within 28 km grid cells. Each point represents the location of a State Capital.

The relation between forest loss and rainfall presented here is correlational. Recently study point out to that forest loss causes 4% of droughts, while droughts account for 0.13% of forest loss per millimetre of rainfall reduced in the Amazon biome[12]. To investigate the possible influence of a drier climate on deforestation in SAB, we superimposed data of Staal et al.[12] on the map of our study region. We found that only 12 cells of a total ≈ 86 non-null cells (14% of our study region) show a statistically significant effect that more severe forest loss tends to take place in drier (or drying) parts (Supplementary Figure 11; Figure was created using data from Staal et al., 2020[12]). We also found for the few cells that have statistical significance ($\alpha = 0.05$) that this effect is ≈ 0.09 % yr^{-1} mm^{-1} in our study region, i.e. near zero. In other words, only in these few areas forest loss may significantly increase (though very little according to the authors' own results) as climate becomes drier. Furthermore, the same authors point out that forest loss has intensified dry seasons especially in the southwestern Amazon (our study region), stressing that the causality of forest loss on rainfall in our study region is more important than the other way around. Additionally, empirical evidence that the length of the rainy season decreases ≈ 0.9 ± 0.34 day per each 10% of forest loss has been published recently[13].

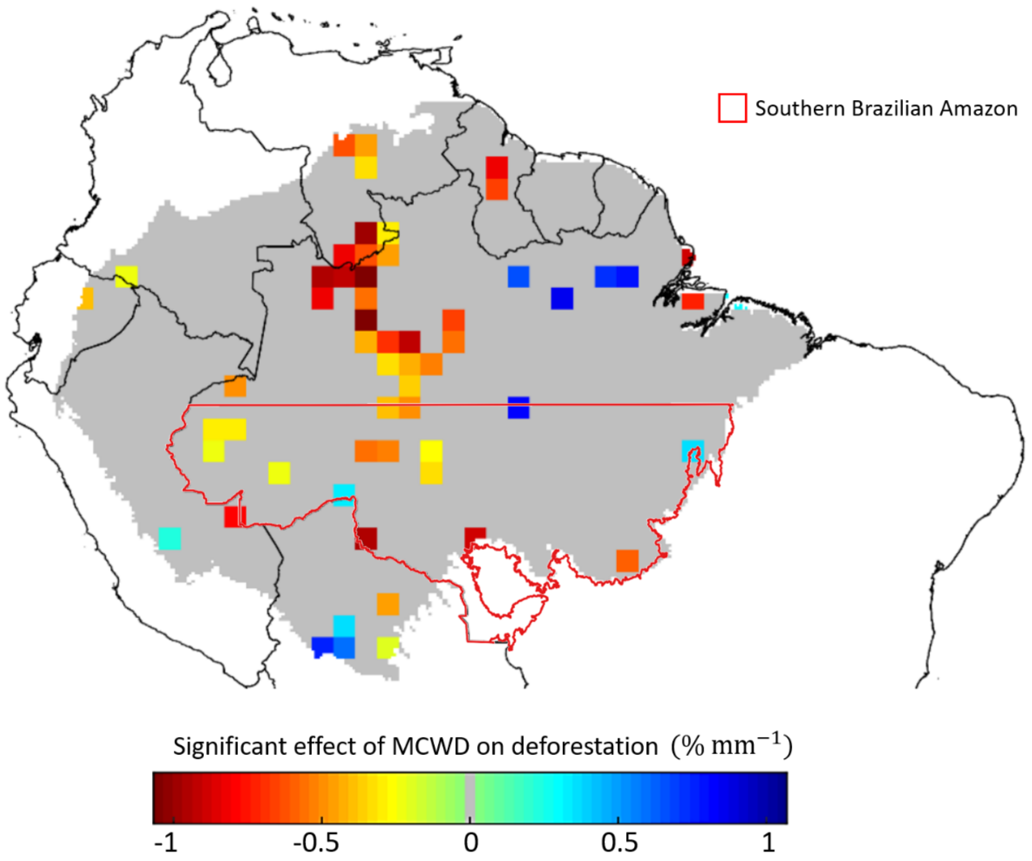

Supplementary Figure 11. **Significant effect of Maximum cumulative water deficit on deforestation.** One degree grid cells with a significant (α = 0.05) temporal effect of the Maximum Climatological Water Deficit on deforestation (MCWD; % yr^{-1} mm^{-1}). Colors indicate change in deforestation for each mm reduction in MCWD as a percentage of the average annual local forest suppression. Note that cells with statically significant effect concentrate in the highly humid forests of Northwestern Amazon.

2.3. Spatially-explicit signal of the reduction of rainfall in the region

We tested whether the mean annual rainfall in Southern Amazon is different between two periods: 1999-2009 and 2010-2019, using Two-Sample t-test. Results are significant at 5% level of significance (Table S8). As a result, Supplementary Figure 13 depicts a clear signal of rainfall reduction, mostly notable where deforestation concentrates in Rondônia and Eastern Amazon.

Supplementary Table 8 | Two-Sample t test with equal sample sizes and unequal variance of annual rainfall means for the 1999–2009 and 2010–2019 periods.

µ 1999–2009	µ 2010–2019	S^2 1999–2009	S^2 2010–2019	t_{calc}	$P_{1\text{-tailed}}$
2092.6 mm	1828.1 mm	113.22 mm	121.86 mm	0.162	0.213

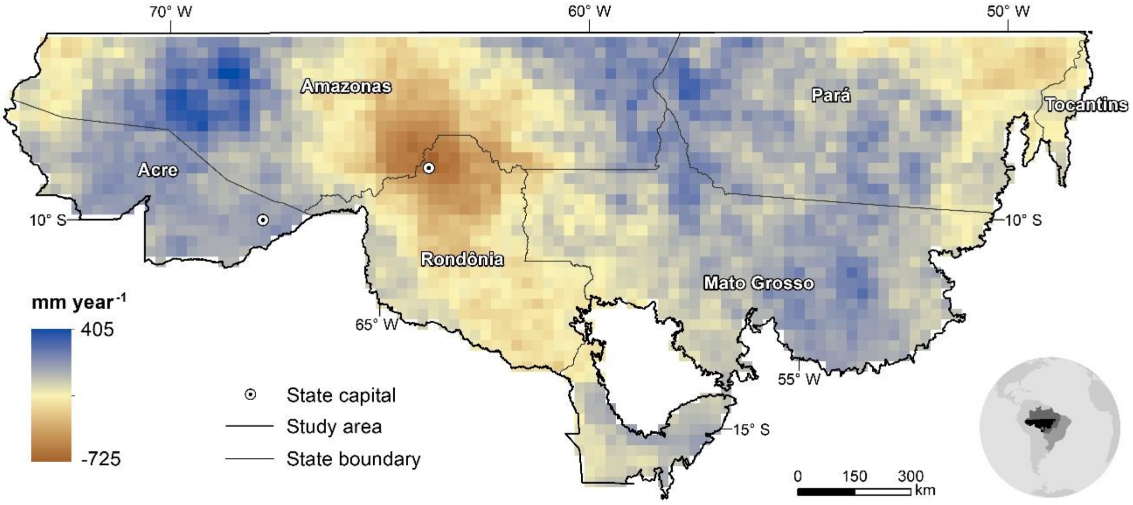

Supplementary Figure 12. Difference of mean annual rainfalls between two periods: 1999-2009 and 2010-2019.

4. SUPPLEMENTARY FIGURES

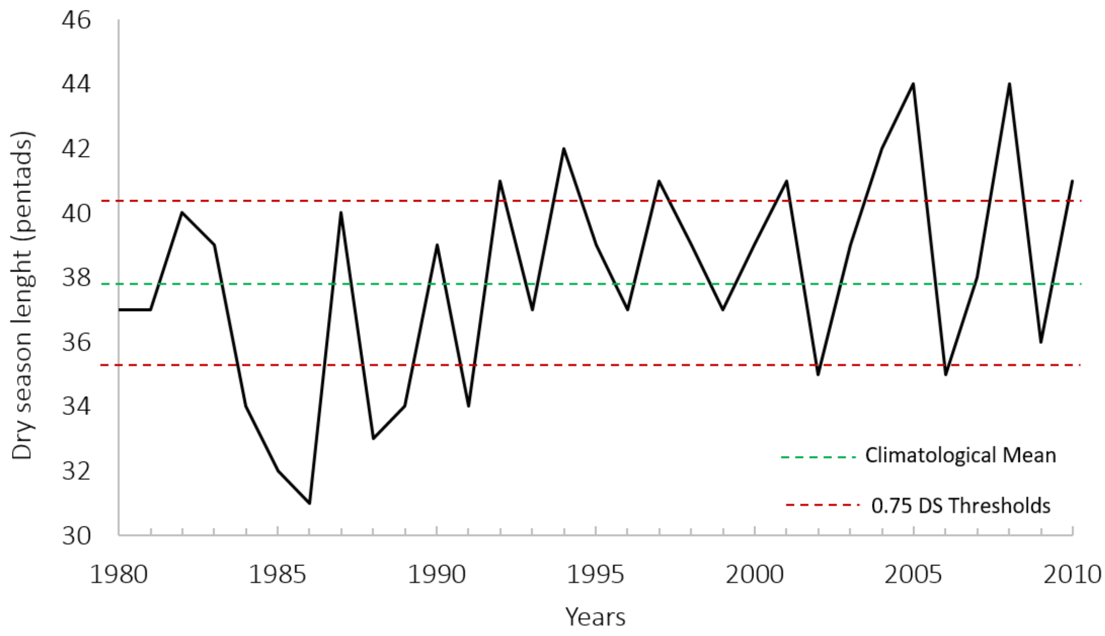

Supplementary Figure 13. **Length of the dry season in Southern Amazon**. Length of the dry season in Southern Amazon between 1980 and 2010 obtained from National Oceanic and Atmospheric Administration/US (NOAA) Climate Diagnostics Center (CDC) daily precipitation gridded data product, version SA24 data. Years are classified into Long dry season (LDS) and short dry season (SDS) using a 0.75σ threshold.

Supplementary Figure 13 was created using data from Agudelo et al. (2019)[14].

5. SUPPLEMENTARY REFERENCES

1. Dancey C. & Reidy J. Statistics without Maths for Psychology: using SPSS for Windows. (Prentice Hall, London, 2004).

2. Rochedo, P. R. R., Soares-Filho, B., Schaeffer, R., Viola, E., Szklo, A., Lucena, A. F. P., Koberle, A., Davis, J. L., Rajão, R. & Rathmann, R. The threat of political bargaining to climate mitigation in Brazil. Nature Climate Change **8**, 695-698 (2018).

3. Strand, J., Soares-Filho, B. S., Costa, M. H., Oliveira, U., Ribeiro, S. M. C., Gabrielle, P., Oliveira, A., Rajão, R., May, P., Hoff, R., Siikamäki, J., Seroa da Motta, Ronaldo R., & Toman, M. Spatially explicit valuation of the Brazilian Amazon Forest's Ecosystem Services. Nature Sustainability **1**, 657–664 (2018).

4. Pires, G., Abrahao, G., Brumatti, L., Oliveira, L., Costa, M., Liddicoat, S., Kato, E. & Ladle, R. Increased climate risk in Brazilian double cropping agriculture systems: Implications for land use in Northern Brazil. Agricultural and Forest Meteorology **228–229,** 286-298 (2016).

5. Ministério da Agricultura, Pecuária e Abastecimento (MAPA). Projeções do Agronegócio 2018/19 a 2028/29, Brasília, Distrito Federal (2019).

6. Batista, E., Soares-Filho B., Barbosa F. A., Merry F., Davis J., Hoff R. & Rajão R. Large-scale pasture restoration may not be the best option to reduce greenhouse gas emissions in Brazil. Environmental Research Letters, **14,** 125009 (2019).

7. Instituto Nacional de Pesquisas Espaciais (INPE). PRODES – Monitoramento da Floresta Amazônica Brasileira por Satélite. INPE database. http://www.obt.inpe.br/prodes/index.php (2019).

8. Cohn, A. S., VanWey, L. K., Spera, S. A. & Mustard, J. F. Cropping frequency and area response to climate variability can exceed yield response. Nature Climate Change **6**, 601–604 (2016).

9. Abrahão, G. M., & Costa, M. H. Evolution of rain and photoperiod limitations on the soybean growing season in Brazil: the rise (and possible fall) of double cropping systems. Agricultural and Forest Meteorology **256–257**, 32–45 (2018).

10. Cramér, H. Mathematical Methods of Statistics Ch. 21 (Princeton University Press, Princeton, 1946).

11. Spearman, C. The Proof and Measurement of Association between Two Things. The American Journal of Psychology **15**, 72–101 (1904).

12. Staal A., Flores B., Aguiar A. P., Bosmans J., Fetzer I. & Tuinenburg O. Feedback between drought and deforestation in the Amazon. Environmental Research Letters **15**, 044024 (2020).

13. Leite-Filho, A. T., Costa, M. H. & Rong F. The southern Amazon rainy season: The role of deforestation and its interactions with large-scale mechanisms. International Journal of Climatology **40**, 2328-2341 (2019).

14. Agudelo, J., Arias, P., Vieira, S. & Martinez, A. Influence of longer dry seasons in the Southern Amazon on patterns of water vapor transport over northern South America and the Caribbean. Climate Dynamics **52**, 2647-2665 (2019).
